# Precision of Myocardial Blood Flow and Flow Reserve Measurement During CZT SPECT Perfusion Imaging Processing: Intra- and Interobserver Variability

**DOI:** 10.2967/jnumed.122.264454

**Published:** 2023-02

**Authors:** Matthieu Bailly, Frédérique Thibault, Gilles Metrard, Maxime Courtehoux, Denis Angoulvant, Maria Joao Ribeiro

**Affiliations:** 1 Nuclear Medicine Department, CHR Orleans, Orleans, France;; 2 UMR 1253, iBrain, Université de Tours, INSERM, Tours, France;; 3 Nuclear Medicine Department, CHRU Tours, Tours, France;; 4 Cardiology Department, CHRU Tours, Tours, France; and; 5 EA4245 T2i, Tours University, Tours, France

**Keywords:** myocardial blood flow, myocardial flow reserve, CZT SPECT, variability

## Abstract

The aim of this study was to evaluate the reproducibility of myocardial blood flow (MBF) and myocardial flow reserve (MFR) measurement in patients referred for dynamic SPECT. **Methods:** We retrospectively analyzed patients referred for myocardial perfusion imaging. SPECT data were acquired on a cadmium zinc telluride–based pinhole cardiac camera in list mode using a stress (251 ± 15 MBq)/rest (512 ± 26 MBq) 1-d ^99m^Tc-tetrofosmin protocol. Kinetic analyses were done with software using a 1-tissue-compartment model and converted to MBF using a previously determined extraction fraction correction. MFR was analyzed and compared globally and regionally. Motion detection was applied, but not attenuation correction. **Results:** In total, 124 patients (64 male, 60 female) were included, and SPECT acquisitions were twice reconstructed by the same nuclear medicine board-certified physician for 50 patients and by 2 different physicians for 74. Both intra- and interobserver measurements of global MFR had no significant bias (−0.01 [*P* = 0.94] and 0.01 [*P* = 0.67], respectively). However, rest MBF and stress MBF were significantly different in global left ventricular evaluation (*P* = 0.001 and *P* = 0.002, respectively) and in the anterior territory (*P* < 0.0001) on interuser analysis. The average coefficient of variation was 15%–30% of the mean stress MBF if the analysis was performed by the same physician or 2 different physicians and was around 20% of the mean MFR independently of the processing physician. Using the MFR threshold of 2, we noticed good intrauser agreement, whereas it was moderate when the users were different (κ = 0.75 [95% CI, 0.56–0.94] vs. 0.56 [95% CI, 0.36–0.75], respectively). **Conclusion:** Repeated measurements of global MFR by the same physician or 2 different physicians were similar, with an average coefficient of variation of 20%. Better reproducibility was achieved for intrauser MBF evaluation. Automation of processing is needed to improve reproducibility.

## Visual Abstract

**Figure:**
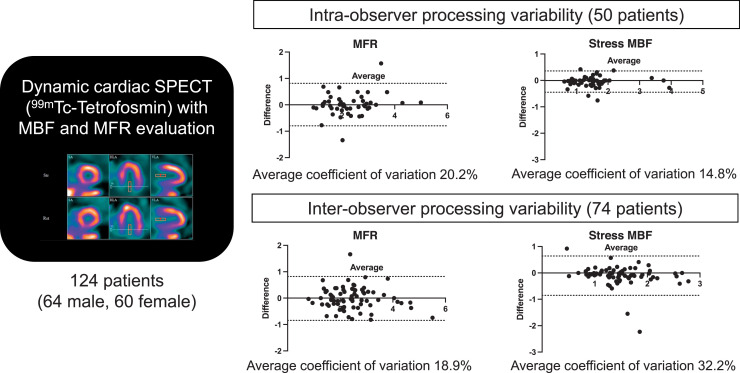

Myocardial blood flow (MBF) at stress (sMBF) and at rest (rMBF) and myocardial flow reserve (MFR) derived from PET perfusion imaging have been shown to provide diagnostic (1*,*^2^) and prognostic (3) information in addition to that provided by relative perfusion analysis alone. Several studies have shown that clinical measurement of MBF and MFR using dynamic cadmium zinc telluride (CZT) SPECT myocardial perfusion imaging with ^99m^Tc-radiopharmaceuticals is technically possible, resulting in an MFR similar to that of PET (4–9).

However, with the idea of greater clinical use, there is a need to evaluate the precision and reproducibility of this measurement. A day-to-day test–retest precision study using a dedicated cardiac camera on a group of 30 patients found that the SD for the difference in measured MBFs was around 30%, including physiologic and processing variability (10). A recent simulation study evaluated the impact of SPECT MFR imprecision on confidence in clinically relevant categorization. The authors concluded that current SPECT MFR precision as categorization with high confidence (>80%) was achieved only for extreme MFRs (<1.0 or > 2.5), with correct classification in only 15% of patients in a typical lab with an MFR of 1.8 ± 0.5 (11). A third paper evaluated the intra- and interobserver repeatability of MBF and MFR values obtained by the same operator and 2 independent operators for 57 patients. This study showed reproducibility that was quite good in the whole-myocardium, left-anterior-descending-artery (LAD), and left-circumflex vascular territories but poor in the right-coronary-artery (RCA) territory (12).

In this study, we evaluated the intra- and interuser processing repeatability of global and regional SPECT MBF and MFR in a larger cohort of patients.

## MATERIALS AND METHODS

### Patient Population

From October 2018 to January 2021, 128 patients referred to 2 nuclear medicine departments for SPECT myocardial perfusion imaging with MBF and MFR quantification were initially enrolled in the CFR-OR trial for coronary artery disease screening (13) (clinicaltrials.gov identifier NCT03586492), and their images were retrospectively reconstructed and analyzed. The study protocol was approved by the institutional review board, and the procedures were in accordance with the Declaration of Helsinki. Every patient gave written informed consent.

**FIGURE 1.**
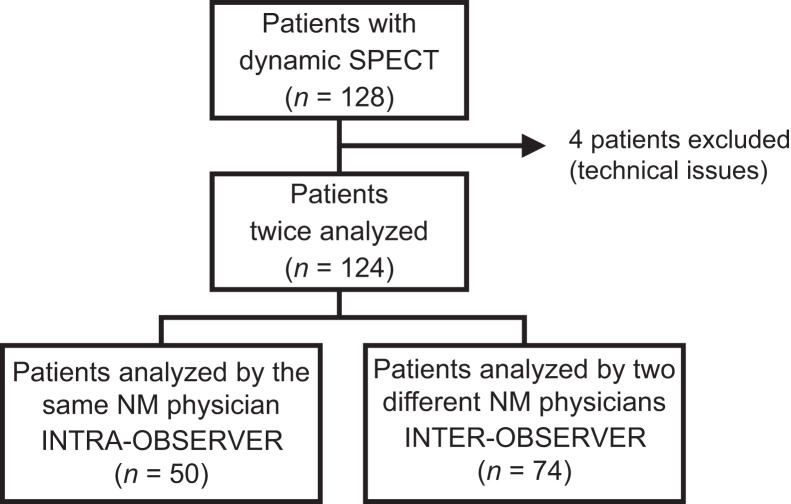
Study flowchart.

The inclusion criterion was dynamic SPECT myocardial perfusion imaging. Exclusion criteria included missing files for new processing or technical issues. Technical issues were reported for MBF and MFR measurement in 4 patients (late acquisition after injection). A flowchart of the study is displayed in Figure 1.

### SPECT Acquisition

List-mode acquisitions were performed on 2 Discovery NM530c cardiac CZT cameras (GE Healthcare) (1 scanner for each department). An initial injection of 37 MBq of ^99m^Tc-tetrofosmin was used to center the patient’s heart in the field of view. Pharmacologic stress was then performed using either a regadenoson (400 μg) injection or a dipyridamole perfusion (0.56 mg/kg), immediately followed by a 250-MBq injection of ^99m^Tc-tetrofosmin at the hyperemia peak and flushing with 50 mL of saline to ensure consistent delivery of a tight bolus. The rest dynamic acquisition was realized 3 h later, with a 500-MBq injection of ^99m^Tc-tetrofosmin.

### SPECT MBF and MFR Quantification

Dynamic SPECT images were reconstructed using Corridor 4DM software (INVIA) on a Xeleris workstation (GE Healthcare). The SPECT initial list-mode data were resampled into frames of 12 × 10 s and 8 × 30 s. The partial-volume value was set to 0.6; the correction factor for myocardial density was set to 1. Spillover of activity from the myocardium to the blood pool was assumed negligible and was set to 0. The uptake rate, *K*_1_, was related to MBF using the Renkin–Crone equation according to Leppo and Meerdink (14), applying a net retention model in which *A* = 0.874 and *B* = 0.443:

$$K_{1}=\text{MBF}\times (1-A\times e^{-\frac{B}{\text{MBF}}}).$$

Residual activity subtraction on rest-image sets after the stress dose was always applied. Because our previous results (15) showed no difference in MFR whether attenuation correction was applied or not, we did not apply it in this study. All MBF and MFR values are presented without attenuation correction. Motion was detected for each patient, and the operator could choose whether to perform motion correction. However, none of the movement was significant, resulting in no correction of data. Double-product (heart rate × blood pressure) correction was used for MBF correction in all studies.

Images of all 124 patients were reconstructed and analyzed by the same expert nuclear medicine physician for the second analysis. The images of 50 patients had initially been reconstructed by the same physician (i.e., intrauser analysis). The images of 74 patients had been analyzed at the first reading by another nuclear medicine physician (i.e., interuser analysis). The mean elapsed time between the 2 analyses was 12.8 mo.

When available, results from invasive coronary angiography were collected. Coronary angiograms were visually assessed by the experienced interventional cardiologist responsible for the procedure. The angiograms were assessed according to the clinical routine, considering available clinical data and patient history. According to the recent guidelines defining very high-risk patients as in need of secondary prevention intervention, we considered all patients having significant coronary artery plaque ≥50% according to the angiographer conclusion (16). We put into perspective the MFR variability by considering the results of invasive coronary angiography, globally and regionally, for each vessel with a significant lesion.

### Statistical Analysis

Continuous variables are presented as mean ± SD. Categoric variables are provided as total number and percentage. Gaussian distribution was assessed using the D’Agostino–Pearson normality test. In analyzing differences between 2 groups, we applied the independent-samples *t* test when comparing continuous variables and the χ^2^ or Fisher exact test, as appropriate, when comparing categoric variables. In analyzing differences between 2 paired groups, the Wilcoxon matched-pairs signed-rank test was applied because of the nonnormally distributed variables. Spearman correlation coefficients were computed between variables. Bland–Altman analysis was used to calculate the bias and the limits of agreement. Precision between the 2 measurements was determined as the coefficient of variation (COV) in the measured difference (COV = SD of the percentage difference). The strength of the agreement between users was evaluated using Fleiss κ. A *P* value of less than 0.05 was considered statistically significant. All analyses were performed using Prism, version 9 (GraphPad).

## RESULTS

**FIGURE 2.**
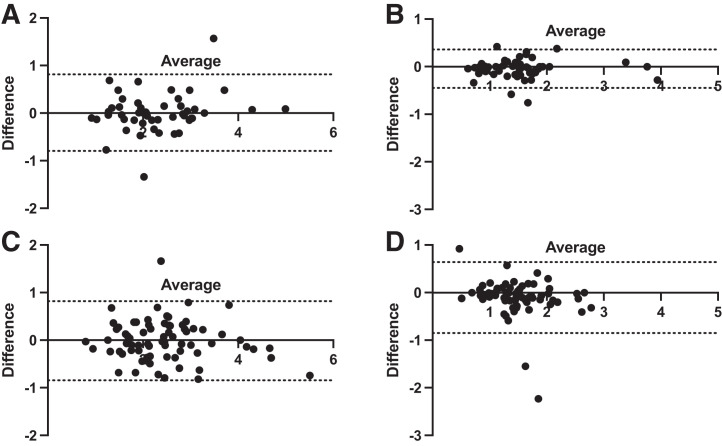
Differences in repeated measurements of MFR and sMBF for intraobserver analysis (A and B, respectively) and interobserver analysis (C and D, respectively). Dashed lines indicate 95% confidence limits. Results are displayed for global left ventricle; results for regional analysis were similar.

The study had 124 patients (61 male, 63 female); both subpopulations were comparable in sex, age, body mass index (BMI), cardiovascular risk factors, and technical parameters (Table 1). Both intra- and interobserver measurements of global MFR had no significant bias (−0.01 [*P* = 0.94] and 0.01 [*P* = 0.67], respectively) (Table 2). Regarding regional MFR, no significant difference was found either for intra- or interobserver analysis. On the interuser analysis, sMBF was significantly different in the global left ventricular evaluation (*P* = 0.0002) and in the anterior territory (LAD) (*P* < 0.0001); rMBF was also significantly different. Lower differences were found for intrauser sMBF evaluation; only sMBF LAD was significantly different (*P* = 0.04). Considering rMBF, no significant difference was found for intraobserver analysis (*P* = 0.15). Bland–Altman analysis showed that the variation in the difference between repeated analyses was consistent across the range of sMBF and MFR considered (Fig. 2).

**TABLE 1.** Patient Description

Parameter	Total	Intraobserver	Interobserver	*P*
Number of patients	124	50	74	
Sex				0.72
Male	61 (45%)	26 (52%)	35 (47%)	
Female	63 (55%)	24 (48%)	39 (53%)	
Age (y)	68 ± 9.3 (41–87)	69 ± 8.6 (41–87)	67 ± 10.5 (44–85)	0.99
BMI (kg/m^2^)	28.3 ± 5.4 (15–44)	28.2 ± 5.5 (18–40)	29.4 ± 6.8 (15–44)	0.33
Stress activity (MBq)	261 ± 14 (240–294)	262 ± 13 (248–294)	258 ± 15 (240–287)	0.99
Rest activity (MBq)	519 ± 18 (468–545)	517 ± 17 (468–538)	522 ± 18 (478–545)	0.99
Positioning activity (MBq)	41 ± 5 (34–55)	41 ± 3 (38–53)	40 ± 5 (34–55)	0.99
CVR factors				
Diabetes	44 (35%)	17 (33%)	27 (36%)	0.87
Hypertension	84 (68%)	35 (70%)	49 (66%)	0.75
Smoking	61 (49%)	26 (51%)	35 (47%)	0.88
Dyslipidemia	82 (66%)	32 (64%)	50 (68%)	0.84
Family history of coronary artery disease	18 (15%)	8 (16%)	10 (14%)	0.92
Mean number of CVR factors	2.3 ± 1 (0–5)	2.4 ± 1 (0–5)	2.2 ± 0.8 (0–5)	0.71

CVR = cardiovascular risk.

Qualitative data are number and percentage; continuous data are mean and range.

**TABLE 2.** Differences in MFR and sMBF Between 2 Measurements, with Statistical Results

Parameter	Mean value ± SD		Mean difference	COV	*P*	Spearman r	Agreement (Bland–Altman)	
	Measurement 1	Measurement 2					Bias	95% limits of agreement
Intraobserver (*n* = 50)								
sMBF LAD	1.72 ± 0.74	1.79 ± 0.73	−0.06	15.1%	0.04	0.86	−0.06	−0.52 to 0.39
sMBF LCx	1.44 ± 0.62	1.48 ± 0.63	−0.04	16.8%	0.22	0.86	−0.04	−0.47 to 0.39
sMBF RCA	1.29 ± 0.75	1.31 ± 0.74	−0.02	13.9%	0.38	0.93	−0.02	−0.38 to 0.34
sMBF global	1.51 ± 0.68	1.56 ± 0.68	−0.04	14.8%	0.10	0.87	−0.04	−0.45 to 0.36
rMBF global	0.72 ± 0.34	0.75 ± 0.41	−0.04	18.8%	0.15	0.87	−0.04	−0.40 to 0.33
MFR LAD	2.41 ± 0.84	2.39 ± 0.98	0.01	20.9%	0.64	0.90	0.01	−0.85 to 0.87
MFR LCx	2.34 ± 0.92	2.37 ± 0.97	−0.04	22.0%	0.79	0.84	−0.04	−0.96 to 0.88
MFR RCA	2.11 ± 0.95	2.10 ± 0.94	0.00	20.0%	0.94	0.88	0.003	−0.80 to 0.81
MFR global	2.29 ± 0.81	2.29 ± 0.89	−0.01	20.2%	0.94	0.88	0.01	−0.80 to 0.81
Interobserver (n = 74)								
sMBF LAD	1.58 ± 0.56	1.78 ± 0.63	−0.21	23.1%	<0.0001	0.72	−0.21	−1.01 to 0.59
sMBF LCx	1.48 ± 0.49	1.54 ± 0.56	−0.06	22.2%	0.53	0.79	−0.06	−0.90 to 0.78
sMBF RCA	1.14 ± 0.48	1.18 ± 0.48	−0.04	21.8%	0.17	0.83	−0.04	−0.56 to 0.48
sMBF global	1.42 ± 0.48	1.52 ± 0.56	−0.11	32.2%	0.002	0.75	−0.11	−0.85 to 0.64
rMBF global	0.62 ± 0.25	0.68 ± 0.27	−0.05	25.3%	0.001	0.77	−0.05	−0.46 to 0.35
MFR LAD	2.45 ± 0.98	2.45 ± 0.93	0.01	20.7%	0.87	0.82	−0.01	−0.88 to 0.86
MFR LCx	2.68 ± 1.12	2.65 ± 1.12	0.03	18.8%	0.40	0.86	0.03	−1.00 to 1.07
MFR RCA	2.33 ± 1.06	2.28 ± 0.97	0.05	19.4%	0.51	0.89	0.04	−0.74 to 0.83
MFR global	2.46 ± 0.94	2.45 ± 0.90	0.01	18.9%	0.67	0.84	−0.01	−0.84 to 0.82

LCx = left circumflex.

MBF data are in mL/min/g.

Bland–Altman analysis of the intrauser evaluation also showed better precision in MBF evaluation (Fig. 2B). The COV between MFR measurements was similar both for intrauser and for interuser evaluations: respectively, 20.2% versus 18.9% for global left ventricular MFR. This COV was similar, at around 20% for all regional MFR territories and analyses; however, the COV was significantly lower for MBF evaluation on the intraobserver analysis than on the interuser analysis: 14.8% versus 32.2% for global sMBF (*P* < 0.001). For the intraobserver subpopulation, 17 patients had a BMI of more than 30, and for the interobserver subpopulation, 27 patients had a BMI of more than 30. Obesity did not impact COV: 21.4% for a BMI of less than 30 and 17.9% for a BMI of more than 30 for intraobserver analysis; 17.3% for a BMI of less than 30 and 22.5% for a BMI of more than 30 for interobserver analysis.

Using an MFR threshold of 2, we noticed good agreement when the 2 measurements were made by the same physician, with consistent classification of 27 patients with an MFR of more than 2 and 17 patients with an MFR of less than 2 (88% of observed agreements; κ = 0.75; 95% CI, 0.56–0.94). Among the 6 patients differently classified, 4 patients had a very similar result of around 2, with a difference of less than 0.2 (1.89 and 2.01, for example). However, this agreement became moderate when the users were different (κ = 0.56; 95% CI, 0.36–0.75), with consistent classification of 41 patients with an MFR of more than 2 and 18 patients with an MFR of less than 2 (79.73% of observed agreements). Fifteen patients were classified differently, with only 2 patients having similar MFR results of around 2 and a difference of less than 0.2.

Thirty-four patients underwent invasive coronary angiography within 3 mo. Seven patients had no significant lesion; 4 of them had global and regional MFRs of more than 2 on both analyses. The other 3 had an MFR of less than 2 on both analyses. Among the 27 patients with lesions, 55 significant plaques were found (24 in the LAD coronary artery, 15 in the left circumflex coronary artery, and 16 in the RCA). Seven of these 55 vessel lesions (12.7%) had discrepant MFRs: 1 below 2 and 1 above, with a mean difference of 0.43 (0.34, 0.93, and 0.04 in the LAD coronary artery, left circumflex coronary artery, and RCA territories, respectively).

## DISCUSSION

In this study, SPECT sMBF and MFR remained globally similar between different measurements whether the analysis was performed by the same physician or by 2 different physicians, except for sMBF (global left ventricular and LAD territory) and rMBF, for which significant differences were found for interuser evaluation. Using the MFR threshold of 2, we found good agreement when the analysis was performed by the same user.

With the development of CZT heart-dedicated SPECT systems, SPECT MBF and MFR have been shown to have a certain diagnostic value for patients with suspected or known CAD and represent a useful supplement to the conventional qualitative diagnostic methods (13*,*^17^*,*^18^). As in PET, an MFR of more than 2 has been considered a normal value, resulting in a very low rate of cardiac events (3*,*^19^). Recent studies have evaluated the day-to-day test–retest precision of sMBF and MFR. Using ^82^Rb PET, the test–retest methodologic precision of serial quantitative global myocardial perfusion for minutes apart is ±10% (mean difference in SD of ±0.09 mL/min/g at rest and ±0.23 mL/min/g at stress) and for days apart is ±21% (mean difference in SD of ±0.2 mL/min/g at rest and ±0.46 mL/min/g at stress), reflecting added biologic variability (20). Recently, Wells et al. determined the day-to-day test–retest precision of SPECT global MBF and MFR to be between 28% and 31% and 33% to 38%, respectively, considering all the processing approaches (use of attenuation correction or not, use of manual motion correction or not) (10). The day-to-day test–retest precision in their study included not only methodologic variability but also physiologic variability in the patient imaged during 2 separate sessions multiple days apart. Though this study reported both methodologic and physiologic variation, the authors noticed a higher variability for SPECT evaluation. Wells et al. advanced the following hypothesis to explain this greater variation: the low extraction fraction of tetrofosmin, the greater statistical noise in the dynamic images, and reduced resolution compared with PET, with the last of these leading to increased partial-volume effects and a need for larger spillover corrections, as well as the additional variability introduced from the manual registration of externally acquired CT images when attenuation correction was applied (because most heart-dedicated CZT SPECT systems are not hybrid).

The impact of attenuation correction and motion correction on MBF accuracy has been evaluated previously by Wells et al. (21). They agreed that attenuation correction had only a small benefit, which may have been offset by the variability due to manual registration of the attenuation map. In our study, we did not apply attenuation correction because it has been our experience, like other investigators, that attenuation correction does not affect MFR (7*,*^15^*,*^21^) and because attenuation correction may not be routinely achievable in that most CZT SPECT cameras are not equipped with CT. Regarding motion correction, we evaluated on a case-by-case basis the need for manual registration, but no correction was needed.

Our study focused only on processing variability (not on physiologic individual variability). We reported a lower SPECT MFR COV of around 20% than did Wells et al. (33%–38% (10)), who evaluated both physiologic and processing variation. A previous study focusing on analysis only, with the same initial dynamic image series on a conventional dual-head camera with sestamibi SPECT MBF using FlowQuant software (Ottawa Heart Institute), reported the SD of the differences to be around 0.30 mL/min/g, with an average MBF of 1.5 mL/min/g, giving a COV of 20% (22). However, we noticed a significantly lower COV on rMBF and sMBF measurements when the processing was performed by the same physician (18.8%–14.8% vs. 25.3%–32.2% for intrauser and interuser, respectively). This lower variation was not noticed on MFR, probably because of the ratio, considering that the variability between 2 different users remained the same on sMBF and rMBF reconstructions. Our limits of agreement for global MFR were also very close to the results of a recent simulation study (11).

Regarding regional MFR, unlike Cichocki et al. (12), we did not notice poor repeatability for MBF and MFR in the RCA territory. Indeed, we observed even lower limits of agreement in the RCA territory on Bland–Altman analysis. The COV remained similar. However, we noticed a greater variability in the LAD territory when processing was performed by different physicians. This finding might also be explained by poor automatic orientation of the heart axis during postprocessing. Better automatic heart orientation and introduction of automatic motion correction are likely to drastically improve interobserver repeatability.

There is a need to increase the analytic precision of SPECT MBF and MFR, as integrated assessment of sMBF and MFR helps improve diagnostic performance (23*,*^24^). sMBF is 2.7 mL/min/g in young, healthy subjects (25). Considering a precision of 15% and 32% for intra- and interuser processing, the lower 95% confidence limit would be 1.9 mL/min/g and 1.2 mL/min/g, respectively. This remains a major limitation in the identification of patients with a moderate reduction in sMBF. In a previous study with invasive coronary angiography correlation, we identified the best sMBF SPECT threshold to be around 1.28 mL/min/g (13). In their simulation study, Renaud et al. showed correct classification in up to only 34% of patients when true MFR was greater than or equal to 1.5 and less than or equal to 2.0. Categorization with high confidence (>80%) was achieved only for extreme MFRs (<1.0 or >2.5), with correct classification in only 15% of patients with an MFR of 1.8 ± 0.5 (11). Our results showed better agreement when the analysis was performed by the same expert nuclear medicine physician. However, 20% of the patients were classified differently using the MFR threshold of 2 in our interuser analysis of 74 patients. Considering the results of invasive coronary angiography on a smaller scale, only 13% of patients were classified differently on a vessel-based analysis. This result is interesting because it counteracts the 20% variability that we observed in the MFR result. At this time, clinical interpretation should remain cautious for a SPECT global MFR of around 2, and even more for regional MFR.

In fact, MFR variability is higher in SPECT than in PET because many steps of the processing remain manual. SPECT MBF is a promising technique, but further work to improve its precision would enhance its potential clinical value, and there is a need for automation and standardization in the processing and software used. This remains difficult, partly because of the lower SPECT spatial resolution and the artifacts at the edge of the field of view, which make it more difficult for the software to automatically identify the location, size, and orientation of the heart. At this point, automated motion-correction software such as what was recently proposed for PET imaging (26) may reduce variability, as may improvements in image quality provided by more advanced reconstruction approaches (27*,*^28^).

Our study had a major limitation: we did not compare our results with MFR calculated in PET, which remains the gold standard. But, as mentioned before, several studies have shown similar quantification of MBF and MFR using dynamic CZT SPECT myocardial perfusion imaging with ^99m^Tc-sestamibi compared with PET (4–7). Moreover, we focused only on a processing variation, with the same initial dynamic data. To our knowledge, this work represents the largest study focusing on the intra- and interuser variability of dynamic SPECT, with clinical impact.

## CONCLUSION

The precision of sMBF analysis, measured as the SD of the difference in measured sMBF, was between 15% and 30% of the mean sMBF if the analysis was performed by the same nuclear medicine physician or by 2 different nuclear medicine physicians. On the other hand, the precision of MFR analysis was around 20% independently of the processing physician. MFR remained similar between different measurements, both in global left ventricular and in regional artery territories, whether the analysis was performed by the same physician or by 2 different physicians. Regarding the MFR threshold of 2, we noticed good agreement on patient classification when processing was by the same physician, whereas agreement was moderate if this was not the case. However, the limits of agreement seemed to be quite wide regarding the threshold of MFR. Though dynamic SPECT is promising, further work is mandatory to improve its precision and enhance its potential value before it can be widely applied to clinical use. The major key point is a need for automation and standardization in the processing and software used.

## DISCLOSURE

This study was supported by CHR d’Orleans. Matthieu Bailly and Gilles Metrard received honoraria and travel grants from GE Healthcare (from previous and other works). Denis Angoulvant received honoraria and travel grants from Astra Zeneca, MSD, Amgen, Servier, Sanofi, Bayer, BMS, Pfizer, Boehringer, Novartis, and Novo Nordisk (from previous and other works). No other potential conflict of interest relevant to this article was reported.

## Notes

KEY POINTS **QUESTION:** How repeatable are MBF and MFR values measured during dynamic SPECT? **PERTINENT FINDINGS:** In 124 patients who were twice processed, the precision of MFR analysis was around 20% independently of the processing physician; for sMBF, it was 15%–30% if the analysis was performed by the same physician or by 2 different physicians. Nevertheless, we noticed quite good agreement on patient classification. **IMPLICATIONS FOR PATIENT CARE:** Clinical interpretation should remain cautious for a SPECT MFR of around 2. To improve the reliability of this promising technique, there is a need for automation and standardization in the processing and software used.
